# Case report: A *de novo* NSD2 truncating variant in a child with Rauch-Steindl syndrome

**DOI:** 10.3389/fped.2023.1064783

**Published:** 2023-06-07

**Authors:** Qi Yang, Di Gong, Shang Yi, Jingsi Luo, Qinle Zhang

**Affiliations:** ^1^ Guangxi Key Laboratory of Birth Defects Research and Prevention, Guangxi Key Laboratory of Reproductive Health and Birth Defects Prevention, Maternal and Child Health Hospital of Guangxi Zhuang Autonomous Region, Nanning, China; ^2^ Department of Genetic and Metabolic Central Laboratory, Maternal and Child Health Hospital of Guangxi Zhuang Autonomous Region, Nanning, China; ^3^ Department of School Infirmary, Guangxi Minzu University, Nanning, China; ^4^ Guangxi Key Laboratory of Birth Defects and Stem Cell Biobank, Nanning, China

**Keywords:** *de novo*, wolf-Hirschhorn syndrome, NSD2 gene, truncating variants, Rauch-Steindl syndrome

## Abstract

Wolf–Hirschhorn syndrome (WHS) is a rare genetic disorder caused by a heterozygous deletion on chromosome 4p16.3, which is called the WHS critical region (WHSC). The major features of this disorder, including “Greek warrior helmet” facies, delayed growth, intellectual disability, seizures, and skeletal abnormalities, are caused by the combined haploinsufficiency of multiple genes. The WHS candidate 1 (WHSC1) gene, also known as *NSD2*, is located in the WHSC and has been reported to associate with Rauch-Steindl syndrome (RSS,OMIM 619695). RSS is a highly heterogeneous disease characterized by mild developmental delay, prenatal-onset growth restriction, low body mass index, and characteristic facial features distinct from WHS. In this report, using whole exome sequencing (WES), we identified a novel *de novo* heterozygous *NSD2* truncating variant in a 7-year-old Chinese girl with Rauch-Steindl syndrome, including failure to thrive, facial dysmorphisms, developmental delay, intellectual disability, and hypotonia. These findings further support that haploinsufficiency of *NSD2* is necessary for WHS, and molecular genetic testing is more accurate to diagnose these patients. The novel variant uncovered in this study further expands the mutation spectrum of *NSD2*.

## Introduction

Wolf–Hirschhorn syndrome (WHS; OMIM 194190) is a rare genetic syndrome caused by terminal chromosome 4p deletions and is also known as 4p-syndrome. WHS is characterized by typical craniofacial features including a “Greek warrior helmet” appearance of the nose (wide flattened nasal bridge continuing to the forehead), microcephaly, widely spaced eyes, a distinct mouth, a short philtrum, micrognathia, downturned corners of the mouth, and poorly formed ears with pits and tags. Growth restriction, postnatal growth deficiency, hypotonia with muscle underdevelopment, and developmental delay/intellectual disability of variable degrees are observed in all affected patients. Other findings include heart and skeletal defects, seizures, abnormal tooth development, and hearing loss (1,2).

WHS is caused by a deletion in the 4p16.3 region, with deletions shorter than 3.5 Mb associated with a milder phenotype without major malformations, and two WHS critical regions—WHSCR−1 and WHSCR−2—overlap the *WHSC1* gene (3–5). The WHS candidate 1 (*WHSC1*) gene, also known as *NSD2* (OMIM 602952), encodes nuclear receptor-binding set domain protein 2 and play a important role in normal development (6). Recently, 36 novel or *de novo* variants in *NSD2* variants were identified in 36 patients with overlapping yet atypical features of WHS, thus defining a novel WHS-like disorder, namely Rauch-Steindl syndrome (RSS,OMIM 619695) (Supplementary Table S1) (7–12). Patients with RSS exhibit a wide range of mild phenotypic features, with core manifestations of microcephaly, intrauterine growth restriction, facial dysmorphisms, autism, intellectual disability, and low birth weight, feeding difficulties, failure to thrive, short stature, speech delay and muscular hypotonia.In this study, In this study, we identified a novel *de novo* NSD2 gene variant [c.2721delT(p.Asn907Lysfs*5)] in a Chinese girl diagnosed with Rauch–Steindl syndrome. In addition, our study further expands the phenotypic spectrum of WHS and the mutation spectrum of *NSD2*.

## Materials and methods

### Patients

**Figure 1:**
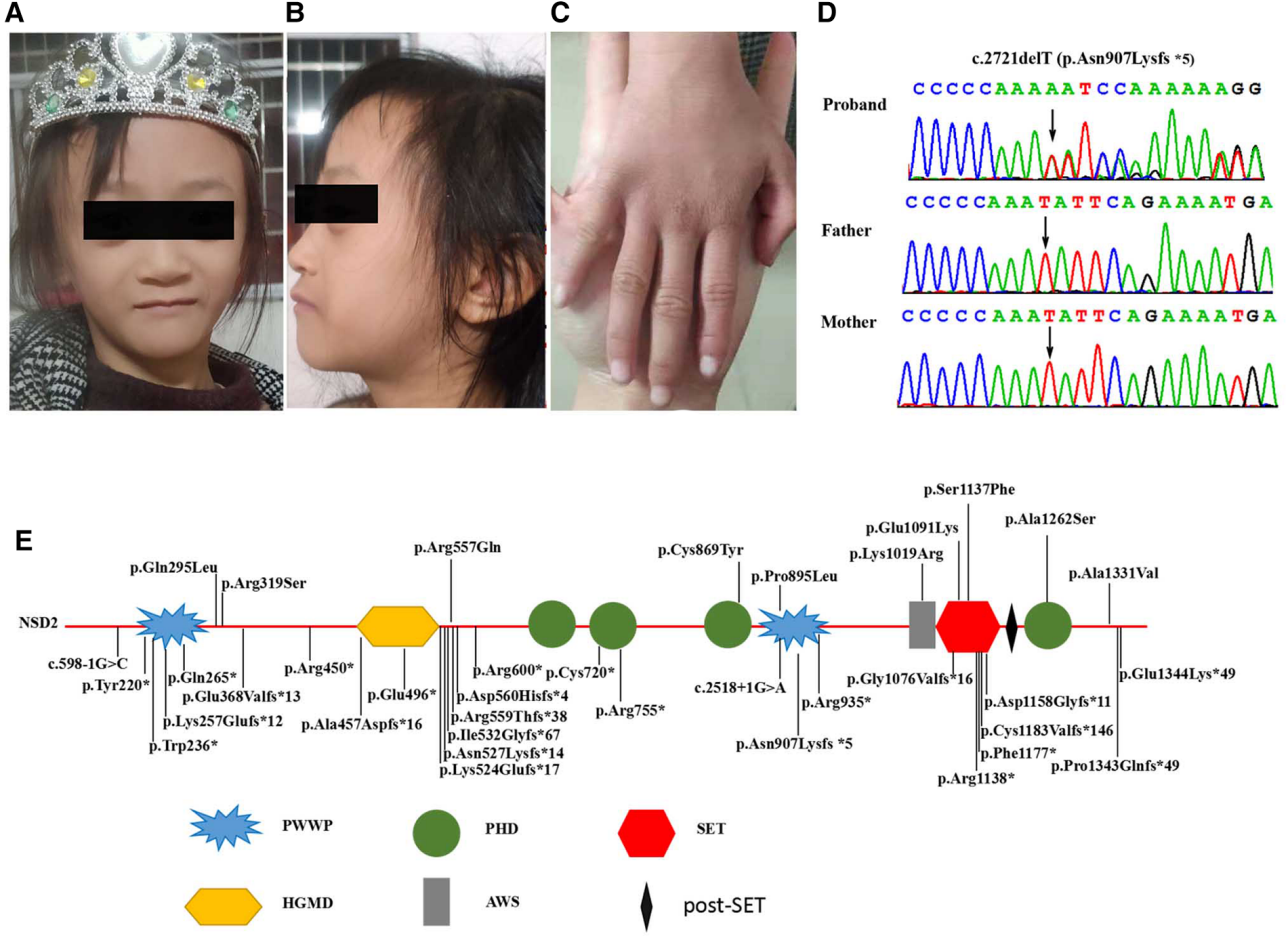
(**A,B**) Clinical photographs of proband showing triangular face, proptosis, hypertelorism, prominent nasal bridge continuing to forehead, micrognathia. (**C**) Mild clinodactyly was observed on her right hand. (**D**) Sequence chromatograms of the *de novo* truncating variant in *NSD2* with their locations indicated by black arrow. (**E**) Schematic diagram of the location of the *NSD2* gene domains and variants. (PWWP, Pro-Trp-Trp-Pro conserved motif; HMG, High mobility group box; PHD, Plant Homeodomain finger; AWS, associated with SET domain; SET, suppressor of variegation, enhancer of zeste, and Trithorax domain).

A 7 year-old-girl was referred to the Genetic Department of Guangxi Maternal and Child Health Hospital for delayed development and growth on November 17th, 2019 (Figure 1). The proband's parents provided written informed consent for the publication of photographs as well as clinical and genetic data. The present study was approved by the Department of Genetic Metabolic Central Laboratory of Guangxi Zhuang Autonomous Region, Women and Children Care Hospital.

### Genetic analysis

#### Whole-exome sequencing and Sanger sequencing

Genomic DNA was extracted from 2-ml peripheral blood samples from the proband. Whole exome sequencing (WES) was performed at the department of Genetic and Metabolic Central Laboratory, Guangxi Maternal and Child Health Hospital. Target DNA was captured using the Agilent SureSelect Human All Exon V5 Kit (Agilent Technologies, Santa Clara, CA, USA) according to the manufacturer's instructions, and the captured libraries were sequenced with the Illumina HiSeq 2500 system (Illumina). The Translational Genomics Expert (TGex) platform (LifeMap Sciences, USA) with the VarElect scoring system was used to annotate the selected variants (13).

#### In silico analysis

SIFT, PolyPhen 2.0, and Mutation Taster software were used to calculate the pathogenicity index of all novel missense variants with unknown clinical significance. The candidate *NSD2* variant identified by WES was confirmed by Sanger sequencing, and its pathogenicity was classified following to American College of Medical Genetics and Genomics/Association for Molecular Pathology (ACMG/AMP) guidelines (14).

## Results

### Clinical description

The proband is a 7 seven-year-old girl born to healthy parents as the first child. Her non-consanguineous parents originated from Guangxi, China. She was admitted to the department of Pediatric Genetic and Metabolic Central Laboratory of Guangxi Maternal and Child Health Hospital due to delayed development and growth. She was born at full term with a birth weight of 2.15 kg and suffered from intrauterine growth restriction during pregnancy. Low birth weight and feeding difficulties kept her hospitalized for a long time. She began walking around 20 months, and spoke her first sentences around 25 months. Growth restriction persisted, she had severe malnutrition [weight: 15 kg (<-3 SD), BMI: 10.7 kg/m^2^], and presented with mild short stature (height: 118 cm, <-1 SD). Her facial dysmorphic features included microcephaly (head circumference: 49 cm, <-2 SD), a triangular face, proptosis, hypertelorism, a broad forehead, a high anterior hairline, short and upslanted palpebral fissures, sparse eyebrows, short philtrum micrognathia, and abnormal teething (Table 1). Dysmorphic features also included small hands and feet, mild clinodactyly of the right hand, and loose skin on the hands and feet. Evaluation using the Chinese Webster Intelligence Scale showed that her full IQ was 85 (Table 1). She had no behavioral issues. The patient's brain MRI, EEG, x-ray of the chest and spine, echocardiography, and abdominal ultrasound examinations were normal, and she experienced no seizures. Her karyotype was determined to be 46, XX.

**Table 1:** Clinical features of the patient with *de novo* NSD2 variant.

Clinical features	Patient
Variants in NSD2 (NM_133330.2)	c.2721delT(p.Asn907Lysfs*5)
Gender	Female
Age at last examination	7 year
Gestation	Full-term
Birth weight	2.15 kg (<-2.5SD)
Birth length	45 cm (<-2SD)
Feeding difficulty	Yes
Muscular hypotonia	Yes
Weight	15 kg (<-3SD)
Height	118 cm (<-1SD)
BMI (kg/m^2^)	10.7
OFC	49 cm (<-2SD)
Developmental delay/intellectual disability/movement delay	Yes; cognitive impairment
Age of walking	20 months
Age of first words	25 months
Brain anomalies	MRI normal
Facial dysmorphisms	Triangular face, proptosis, hypertelorism, broad forehead, high anterior hairline, short and upslanted palpebral fissures, sparse eyebrows, short philtrum micrognathia, and abnormal teething.
Other anomalies	Small hands and feet, mild clinodactyly of the right hand, and loose skin on the hands and feet.

### Mutation analysis

We performed WES for the proband at an average depth of coverage of 20×, and 95.6% of the targeted regions were covered. A total of 132,342 variants or indels were detected in the proband. Variants with a minor allele frequency of >0.01 in any of the variant databases (e.g., 1,000 Genomes, dbSNPand Genome Aggregation Database)were excluded.Using TGex software (LifeMap Sciences, United States), ten candidate variants matched with known phenotypes in ten genes (*FGFR3*, *PCNT*, *CDC45*¸*UBE3B*, *TBCE*, *NSD2*, *CPLX1*, *SETD5*, *NOTCH2*, *FZD2*) were subsequently extracted. The variants in the *PCNT*, *CDC45*, *UBE3B*, *TBCE* and *CPLX* genes were heterozygous. The diseases caused by these genes are autosomal recessive and are therefore excluded. The variants of the *FGFR3*, *SETD*, *NOTCH2* and *FZD2* genes were inherited from the unaffected father or mother, but were found not to be responsible for the phenotype (Supplementary Table S2). Then, a novel *de novo* heterozygous frameshift variant c.2721delT(p.Asn907Lysfs*5) in the *NSD2* gene (RefSeq: NM_133330.2) was identified in the proband (Figure 1D). The variant has not been previously reported in 1,000 Genomes, dbSNP, Genome Aggregation Database and in control databases. According to the ACMG/AMP standards and guidelines for the variant, the novel variant is pathogenic (PVS1, PS2, PM2).

## Discussion and conclusion

*NSD2* (WHSC1) is∼90 kb long and located on human chromosome 4p16.3, and encods the nuclear SET domain-containing transcriptional regulatory protein, which contains four development-related domains: a PWWP domain, an HMG box, a SET domain, and a PHD-type zinc finger. NSD2 is a SET domain histone methyltransferase responsible for the methylation of H3K36, is expressed widely across many tissue types, and participates in a variety of biological processes, including early development, cytokine signaling, the DNA damage response, and class switch recombination. Haploinsufficiency of *NSD2* (pLi = 1.00) is thought to be to be an important part of the mutational mechanism of WHS. Heterozygous knockout-*NSD2* mouse models show growth restriction, craniofacial malformation, and midline fusion defects (15, 16). Zanoni et al. demonstrated that loss-of-function and missense variants in *NSD2* lead to reduced methylation activity and are associated with a distinct developmental phenotype (11). These findings suggest that NSD2-deficiency is responsible for multisystem abnormalities in patients with Rauch–Steindl syndrome. Recent reports have confirmed that patients with novel loss-of-function or missense variants in *NSD2* exhibit a variety of abnormalities including facial dysmorphism, microcephaly, intrauterine and postnatal growth restriction, craniofacial malformations, failure to thrive, intellectual disability, speech delay, behavioral/psychological issues, and skeletal and limb abnormalities (7–12). In the present study, the *de novo* variant c.2721delT is located in the PWWP2 domain in NSD2 and is predicted to create a frameshift starting at codon 907 leading to a stop codon 5 positions downstream (p.Asn907Lysfs*5). This variant may result in the absence of protein production with a significant decrease in mRNA level due to nonsense-mediated decay degradation. The present patient showed common phenotypes associated with Rauch–Steindl syndrome, including facial dysmorphism, microcephaly, a degree of intellectual disability, growth restriction, severe malnutrition, short stature, small hands and feet, muscular hypotonia, mild clinodactyly of right hand, and loose skin on the hands and feet, and these phenotypes are thought to be due to the loss-of-loss-of-function variant detected in *NSD2*.

To date, 37 *NSD2* variants have been identified, including ten missense variants (eight *de novo,* two inherited), fifteen frameshift variants (nine *de novo*, two inherited, four undetermined), ten nonsense variants (nine *de novo*, two undetermined), and two splice site variants (two *de novo*) (Supplementary Table S1). These variants are distributed across the entire gene, and 87.5% (28/32) are *de novo* (Supplementary Table S1, Figure 1). Of the 37 *NSD2* variants, 11 (including our variant) located on the PWWP domain, including two missense variants (two *de novo*), four frameshift variants (three *de novo*, one undetermined), and four nonsense variants (three *de novo*, one undetermined). This is not significant compared to the variants in the entire gene. In addition, Rauch-Steindl syndrome is a highly heterogeneous disease caused by variants in the *NSD2* gene. The phenotypes of the Rauch-Steindl syndrome are complex but several common characteristics are observed in most patients (11). Zanoni et al. have been reported that the core NSD2-associated phenotype includes mostly mild developmental delay, prenatal-onset growth retardation, low body mass index, and characteristic facial features distinct from WHS (11). Patients carrying missense variants were significantly taller and had more frequent behavioral/psychological issues compared with those harboring truncating variants. The results showed that the relationship between genotype and phenotype seemed to be related to the mutation type of the mutant, but not to the position of the mutant in the protein. Limited by the currently reported cases and variants, these results should be seen as provisional. Future studies with the report of increasing numbers of patients will therefore be necessary to to refine the phenotype and clarify genotypic effects and other phenotypic determinants.

Typical WHS has been described as a disorder with special craniofacial features, growth restriction, intellectual disability, seizures and skeletal abnormalities resulting from heterozygous deletions in the 4p16.3 region (1). Compared with typical WHS patients, an atypical mild manifestation was observed in our patient. In addition to our patient, other reported patients showed less marked craniofacial features of WHS. 60%–70% of typical WHS patients have severe skeletal anomalies including kyphosis/scoliosis with malformed vertebral bodies, accessory or fused ribs, clubfeet, and split hands. Only 39% of patients with a *NSD2* variant have skeletal abnormalities, which is less severe than in typical WHS patients (9–11). Mild clinodactyly in the right hand of the present patient was observed. This indicates that the *NSD2* gene may not be the main cause of WHS craniofacial features and skeletal anomalies, and it may have a cumulative effect on severe intellectual disability, typical craniofacial features, and skeletal anomalies of WHS with other genes located in the 4p16.3 region. Seizures are the most common problem in children with WHS (90%–100%) and are the greatest problems in the clinical management of WHS (17). As in previously reported patients with *NSD2* variants, our patient also lacked seizures or seizure-like episodes, further indicating that seizures are not associated with the *NSD2* gene and that other genes located within the 4p16.3 region such as *LETM1* may be responsible for seizures (18). In addition, the severity of the intellectual disability in patients with *NSD2* patients was milder compared with patients carrying the most common 4p deletions (which are between 5 and 18 Mb in length). Furthermore, the majority of individuals with *NSD2* variants lack many of the most common manifestations of WHS, such as orofacial clefts and defects and genital, cardiac, and renal malformations. Therefore, NSD2-deficiency may be necessary for WHS, but it is not sufficient to cause WHS. In addition, genetic diagnosis is the best method for detecting the cause of the syndrome in these patients.

## Conclusions

In conclusion, we found a *de novo* heterozygous frameshift pathogenic variant, c.2721delT(p.Asn907Lysfs*5), in the *NSD2* gene in a Chinese girl with Rauch–Steindl syndrome using WES. Our findings support that this loss-of-function variant in *NSD2* is a cause of a syndrome consisting of intellectual disability and developmental delay. Detailed clinical features and molecular diagnosis will further help our understanding of the phenotype-genotype correlations of *NSD2* pathogenic variants and related disorders including WHS. In addition, molecular genetic testing of NSD2 is a useful tool for clinical diagnosis and genetic counseling of these patients. The novel pathogenic variant uncovered in this study expands the mutation spectrum of *NSD2*.

## Data Availability

The datasets presented in this study can be found in online repositories. The names of the repository/repositories and accession number(s) can be found below: https://www.ncbi.nlm.nih.gov/sra/, PRJNA888339.
